# Hemolytic parasites affect survival in migrating red-tailed hawks

**DOI:** 10.1093/conphys/coac075

**Published:** 2022-12-21

**Authors:** Christopher W Briggs, Kris A Dudus, Teresa E Ely, Laura A Kwasnoski, Cynthia J Downs

**Affiliations:** Golden Gate Raptor Observatory, Golden Gate National Parks Conservancy, Sausalito, CA 94965, USA; Department of Biology, Colgate University, Hamilton, NY 13346, USA; Department of Environmental Biology, State University of New York College of Environmental Science and Forestry, Syracuse, NY 13210, USA; Golden Gate Raptor Observatory, Golden Gate National Parks Conservancy, Sausalito, CA 94965, USA; National Park Service, Gulf Breeze, FL 32563, USA; Golden Gate Raptor Observatory, Golden Gate National Parks Conservancy, Sausalito, CA 94965, USA; Department of Biology, Hamilton College, Clinton, NY 13323, USA; Department of Environmental Biology, State University of New York College of Environmental Science and Forestry, Syracuse, NY 13210, USA

**Keywords:** red-tailed hawk, immune function, H/L ratio, body condition, blood parasites

## Abstract

Migrating birds face a myriad of hazards, including higher exposure to parasites and numerous competing energy demands. It follows that migration may act as a selective filter and limit population growth. Understanding how individual-level physiological condition and disease status scale up to population dynamics through differential survival of individuals is necessary to identify threats and management interventions for migratory populations, many of which face increasing conservation challenges. However, linking individual physiological condition, parasite infection status and survival can be difficult. We examined the relationship among two measures of physiological condition [scaled-mass index and heterophil/leukocyte (H/L) ratio], hematozoa (i.e. hemoparasites) presence and abundance, and constitutive immunity in 353 autumn migrating red-tailed hawks (*Buteo jamaicensis calurus*) from 2004 to 2018. Hematazoa (i.e. *Haemoproteus* and *Leucocytozoon*) were in the blood smears from 139 red-tailed hawks (39.4%). H/L ratio decreased with scaled-mass index. Adults had a significantly higher H/L ratio than juveniles. Our two measures of immune defences, hemolytic-complement activity and bacteria-killing ability, were highly positively correlated. Our most notable finding was a negative relationship between *Haemoproteus* parasitemia and survival (i.e. documented individual mortality), indicating that haemosporidian parasites influence survival during a challenging life stage. The effect of haemosporidian parasites on individuals is often debated, and we provide evidence that parasitemia can affect individual survival. In contrast, we did not find evidence of trade-offs between survival and immune defences.

## Introduction

Migration is an important life-history adaptation for many species, but individuals face a myriad of hazards during migration, including predation (Cimprich and Moore, 1999; Dierschke, 2003), competition for resources (Rappole and Warner, 1976), variable quality of food and food limitation (Moore, 1995), and unfamiliar or variable habitats (Bauer *et al.*, 2020; Brown *et al.*, 2021). Migration risks can lead to high mortality, which subsequently may limit population growth (Owen and Black, 1989; Newton, 2006; Klaassen *et al.*, 2014). For example, migrants may face increased parasite exposure relative to nonmigrants because they cover greater distances, congregate at high densities at stop-over sites while energetically depleted, and are exposed to parasites not present in their winter or summer range (Møller and Szép, 2011). Thus, parasites might act as an additional source of mortality in migrants, creating a strong selective filter (Waldenström *et al.*, 2002; Altizer *et al.*, 2011), particularly at stop-over sites (Krauss *et al.*, 2010). To mitigate this threat on a short timescale, migrants may invest in energetically costly immune defences (Møller and Erritzøe, 1998; Lochmiller and Deerenberg, 2000). For example, immune defence organs are larger in migratory species relative to closely related nonmigratory species (Møller and Erritzøe, 1998). Similarly, birds that disperse longer distances and thus undergo a costly movement have a higher investment in immune defences (Møller *et al.*, 2004; Snoeijs *et al.*, 2004).

Alternatively, given limited energy resources and high energy demand during migration (Gwinner, 2012; Butler, 2016), individuals are likely forced to allocate resources between immune defences and other physiological processes, creating a trade-off between current immune defences and probability of future survival (Sheldon and Verhulst, 1996; Weber and Stilianakis, 2007). For example, three species of thrushes exhibited immunosuppression and reduced fat reserves during spring migration (Owen and Moore, 2006). In Swainson's thrushes (*Catharus ustulatus*), some of this immunosuppression occurred with the onset of migratory restlessness and reflected preparation for migration (Owen and Moore, 2008a). However, once migration began, immunosuppression was exacerbated by the energetic costs of migration (Owen and Moore, 2008b). Understanding how individual-level physiological condition and disease status scale up to population dynamics through differential survival of individuals is necessary to identify threats and management interventions for migratory populations, many of which are facing increasing conservation challenges (Downs and Stewart, 2014; Ohmer *et al.*, 2021).

Understanding the consequences of immune-based trade-offs for population dynamics requires linking them with fitness measures (Graham *et al.*, 2011; Downs *et al.*, 2014). Physiological indices, such as size/weight residuals, stress hormone levels or parasite presence/intensity, are used to measure aspects of an individual's physiological condition, assuming that they are a predictor of an individual's fitness (Sánchez *et al.*, 2018). Condition indices are used, in part, because they can be easier than measuring fitness parameters directly, such as survival or offspring recruitment (Davis *et al.*, 2008; Müller *et al.*, 2011). When validated, measures of physiological condition provide insights into individual quality, a trait that is notoriously difficult to quantify, because physiology mediates life history and fitness traits (Zera and Harshman, 2001; Flatt *et al.*, 2011). However, condition indices are rarely validated with population parameters.

Heterophil/lymphocyte (H/L) ratio and scaled-body mass (aka body condition) are indices used commonly to estimate general physiological condition. A growing body of literature suggests that H/L ratios may be a good proxy for physiological condition in ecological studies of birds (e.g. Saks *et al.*, 2003; Davis *et al.*, 2008; Catitti *et al.*, 2022; Ribeiro *et al.*, 2022). Individuals with naturally higher H/L ratios have a higher risk for infection (Al-Murrani *et al.*, 2002, 2006), and H/L ratio increases in response to parasitic infections (Lobato *et al.*, 2005). H/L ratios have also been associated with measures of individual quality in some systems. For example, higher H/L ratios were negatively associated with growth in nestling pied flycatchers (*Ficefula hypoleuca*) and negatively correlated with body condition in northern goshawks (*Accipiter gentilis*) (Hanauska-Bro and Dufty, 1996). In contrast, they were positively associated with recruitment into the adult population (Lobato *et al.*, 2005; Moreno *et al.*, 2005). Generally, relatively smaller H/L ratios indicate a less stressed individual in good physiological condition (Saks *et al.*, 2003).

Herein, we assess the relationship between multiple metrics of physiological quality that reflect immunocompetence, energy reserves, migration timing and population performance, specifically survival. We examine the relationships among hematozoa infections, investment in immune defences, physiological condition and survival in red-tailed hawks (*Buteo jamaicensis*) caught during the autumn migration season along coastal California, USA. Hematozoan infections are globally distributed and ubiquitous among birds (Garnham, 1966; Valkiunas, 2004; Perkins, 2014). We predict that individuals with greater parasitemia and/or higher H/L ratio would have lower survival due to the associated energetic costs of current or prior exposure to parasites. We also predict that individuals in poorer body condition would have reduced survival because of lesser energy reserves. Constitutive immune defences are the first line of defence against infection (Murphy *et al.*, 2007; Downs and Stewart, 2014). Thus, individuals with greater constitutive immune defences should be better able to respond to new infections and should have higher survival. We used two commonly studied measures of the complement cascade, bacteria-killing ability and hemolytic-complement activity, as our measure of immune defences (French *et al.*, 2010; Demas *et al.*, 2011). Ostensibly, these assays are not affected by previous challenges and the development of pathogen-specific antibodies, and higher values indicate a greater capacity to limit infection (Matson *et al.*, 2005, 2006; Tieleman *et al.*, 2005). Finally, we predict that later migrants, who are often migrating from farther distances (Hull *et al.*, 2009), will have higher energetic demands of their longer migratory journey resulting in a stronger trade-off with lower immune function.

## Methods

### Sample collection

Red-tailed hawks were caught during autumn migration (August–December) of 2004–2018 at the Golden Gate Raptor Observatory in the Marin Headlands, Sausalito, CA, USA (37°50′N, 122°30′W). Individuals were caught using mist nets, dho-ghaza nets and bow-nets as described by Hull *et al.* (2009). Each hawk was banded with a US Geological Survey band, and morphometric data (e.g. weight and wing chord) were collected. Sex of juveniles was determined following Pitzer *et al.* (2008), and sex of adults was determined using Tomalty *et al.* (2016). Up to 1 ml of blood was collected from the medial metatarsal vein using a 25-gauge needle and a 3-cc syringe. Two blood smears were created immediately (Owen, 2011). Blood was stored on ice for less than 4 hr until centrifuged in the laboratory. Plasma was stored frozen at −20°C for up to 12 weeks and then transferred to a −80°C freezer where it was stored until analysed. We calculated the mass index as one measure of body condition by correcting mass with unflattened wing chord for all individuals with empty crops (Peig and Green, 2009, 2010). Individuals without weights or who had any food in the crop (N = 12) were assigned the mean body condition value. Encounters of birds, both living and dead, were reported to the USGS Bird Banding Lab. Upon receipt of the report, we attempted to follow up all encounters to ensure accuracy of the report (e.g. if the bird was alive, how recently it may have died, when the individual was first encountered). Samples were collected under the appropriate state and federal permits, and sample protocols were approved by California State Scientific Collection Permit (permit number 007333) and USGS Bird Banding Lab (permit number 21827).

### Blood smear evaluation

Blood smears were air-dried, fixed in methanol and stained with Giemsa stain for the 2013 samples (Valkiūnas *et al.*, 2008) and Diff-Quick (Richard Allan Scientific) for all other samples. The smears were examined at a magnification of 1000× with oil immersion. We counted the number of heterophils and lymphocytes per 100 fields of a slide. Cells and blood parasites were identified using standard guidelines (Remple, 2004; Zajac and Conboy, 2012; Campbell and Ellis, 2013), and we used the mean from the two slides to obtain an H/L measure for each individual. Parasite (e.g. *Plasmodium*, *Haemoproteus* and *Leucocytozoon*) intensity was calculated by the number of infected cells found per 10 000 red blood cells under 1000× objective. We estimated the number of red blood cells by comparing each field to a set of standardized photographs with known numbers of red blood cells (Ellis *et al.*, 2014). We used the mean number of infected cells from two slides as our measure of parasitemia. Parasites were detected by K.A.D. without any knowledge of H/L ratio or data collected in the field. Birds without infected red blood cells were excluded from the parasite intensity analysis.

### Immune function

To quantify bacteria-killing ability, we followed the procedure developed by French and Neuman-Lee (2012) calibrated for red-tailed hawks. Briefly, we plated 20 μl of a 1:2 dilution of the sample in phosphate-buffered saline (PBS) in duplicate. We also plated a positive control (20 μl of PBS) and negative control (25 μl of PBS) in triplicate. We added 5 μl of 10^5^ bacteria ml^−1^*Escherichia coli* (ATCC #8739, Epower Microorganism from Microbiologics Inc., St. Cloud, MN #0483E7) to each sample and the positive controls. We chose this microbe because it is the most common one used in ecoimmunology studies and it is ecologically relevant (Matson *et al.*, 2005; Tieleman *et al.*, 2005; Demas *et al.*, 2011). The plate was vortexed for 1 min at 500 rpm, and then incubated for 30 min at 37°C. We then added 125 μl of tryptic soy broth to all wells, vortexed the plate for 1 min at 300 rpm and measured for absorbance [as optical density (OD)] at 300 nm on a spectrophotometer. The plate was incubated for 12 hr at 37°C and read again at 300 nm on a spectrophotometer. This first reading served as an internal control for the later growth reading. Bacteria-killing ability was calculated as $\Big(1-\frac{(\mathrm{OD}\ \mathrm{sample}\ \mathrm{end}-\mathrm{OD}\ \mathrm{sample}\ \mathrm{baseline})}{(\mathrm{OD}\ \mathrm{control}\ \mathrm{start}-\mathrm{OD}\ \mathrm{control}\ \mathrm{end})}\Big)\ast 100\%$. Larger values (%) indicate greater constitutive protection as measured by this immune assay.

Hemolytic-complement activity in serum was measured with a method adapted from Sinclair and Lochmiller (2000) and calibrated for red-tailed hawks. Briefly, we diluted serum to a 1:5 and a 1:10 dilution with dextrose gelatin veronal buffer (DGV; catalogue no. 10-539B; Lonza Inc., Allendale, NJ, USA). In a 96-well plate, we mixed 40 μl of each dilution with 25 μl of 0.06% suspension of sheep red blood cells (SRBC; Innovative Research, Novi, MI, USA) in DGV and 25 μl of a 1:40 dilution of rabbit anti-SRBC antibodies (Sigma-Aldrich, St. Louis, MO, USA; product no. S1389) in DGV. We included a 100% lysis control of 65 μl of deionized water and a 0% lysis control of 65 μl DGV. We added 25 μl of diluted SRBC, but no antibodies, to both controls. Samples and controls were analysed in duplicate. The plate was shaken for 5 min at 300 rpm on a plate shaker, and then incubated for 90 min at 37°C. The plate was then centrifuged for 5 min at 500 rpm, and 60 μl of supernatant was transferred to a new 96-well plate. Absorbance as OD was measured at 405 nm. From the OD readings from the two dilutions, we calculated the CH50—the reciprocal of the dilution of serum that will lyse 50% of the SRBC (Mayer, 1948; Sinclair and Lochmiller, 2000); greater values indicate more protection.

**Table 1:** Pearson correlation coefficients (below the diagonal) and Bonferroni-corrected *P*-values (above the diagonal) of measures of red-tailed hawks trapped during migration in the Marin Headlands, California, including age, bacteria-killing ability (BKA), scaled-mass index (SMI), *Haemoproteus* parasitemia (Haemo), heterophil/leucocyte ratio (H/L), *Leucocytozoon* parasitemia and sheep red blood cell lysing ability (SRBC). Bold values indicate significant correlations.

	Age	BKA	SMI	Haemo	H/L	Leuco	SRBC
Age	—	0.93	1	0.72	**<0.001**	1	1
BKA	−0.08	—	0.45	0.83	0.94	1	**<0.0001**
SMI	−0.02	−0.04	—	1	**0.01**	1	1
Haemo	−0.02	−0.01	−0.05	—	1	1	1
H/L	**0.34**	0	**−0.17**	−0.04	—	1	1
Leuco	−0.05	0.04	−0.06	−0.01	−0.01	—	1
SRBC	−0.07	**0.48**	0.05	0	0.02	0.02	—

### Statistical analysis

We log_10_ transformed our measures of parasitemia for all analyses to reduce outlier effects. We examined the relationships between log of parasitemia of *Haemoproteus*, log of parasitemia of *Leucocytozoon*, H/L ratio, age, scaled-mass index, bacteria-killing ability and hemolytic-complement activity using Pearson correlations in R 3.5.2 (R Core Team, 2020) to provide context for the analysis and to assess how different health metrics were related within individuals in this study. We used Bonferonni corrections to account for multiple comparisons.

To measure monthly apparent survival, we used a multistate Burnham joint live/dead models of survival in Program MARK 8.2 (White and Burnham, 1999). An individual was considered encountered in the month it was reported to the Bird Banding Lab. Dead encounters were all confirmed by the finder, and all dead encounters suggested that individuals were recently deceased (e.g. individual was not desiccated, recovery was not of a band only).

We included two states in our modelling: one for when individuals were first captured and one for all subsequent encounters. Transition probability from initial capture to subsequent capture was fixed to 1, and individuals could not transition back to the initial state (i.e. transition back to the original state was fixed to 0). This way, all metrics of body condition and immune function could be applied to the month an individual was trapped and would not influence subsequent survival estimation. Alternatively, we could model if our condition metrics affected survival beyond the first month of capture to subsequent months by applying the covariate to each state.

We also assigned individuals into two groups, adults and juveniles. Juveniles were individuals in first basic plumage. Adults were individuals in second basic plumage; they molt into their adult plumage the summer after they fledge (Pyle, 2008). In our survival modelling, we graduated juveniles into the adult age class the August following their initial capture. We first modelled reporting rate while leaving the remaining model time and age dependent. For reporting rate, we separated individuals trapped before October 1 and after October 1 each year to account for higher probability of individuals migrating from the Intermountain West (Hull *et al.*, 2009), comparing to the null (i.e. no effect) model.

Once we determined the most parsimonious model for reporting rate, we modelled fidelity rate followed by encounter probability. For both encounter probability and fidelity, we singularly examined effects of month, year and age (i.e. juvenile versus adult) as juveniles and adults might have different movement patterns that could affect fidelity to an area and probability an individual is encountered (Morrison and Baird, 2016; McCrary *et al.*, 2019), as well as survival (Newton *et al.*, 2016).

Using the most parsemonious models, we modelled survival probability by examined additive effects of month, year and age, and also univariately examining the effects of six covariates; parasitemia of *Haemoproteus*, parasitemia of *Leucocytozoon*, H/L ratio, bacteria-killing ability, SRBC and scaled-mass index, creating a total of nine survival models. We assigned average values to individuals for which we could not quantify bacteria-killing ability or hemolytic-complement activity due to lack of plasma. We assessed model performance using Akaike information criterion adjusted for small sample size (AIC*_c_*) values (Burnham and Anderson, 2002). To account for model uncertainty, we model-averaged model results within Program MARK to obtain estimates of survival as well as the effects of each parameter. All results were presented as mean ± SE. *α* was set at 0.05 for all tests.

## Results

We captured and collected blood smears from 353 red-tailed hawks, 331 were juveniles and 22 adults. We had a total of 51 encounters (14.4% of birds): 13 encounters of individuals who were alive over the study and recovered 38 dead individuals from this group. Hematazoa were found in 139 red-tailed hawks (39.4%). Of those, 71 individuals had *Haemoproteus* (average log parasitemia, 2.3 ± 19.8 infected cells/10 000 erythrocytes), 86 had *Leucocytozoon* (average log parasitemia, 0.9 ± 3.4 infected cells/10 000 erythrocytes) and no individuals had *Plasmodium*. Neither H/L ratio nor immune defences were correlated with hematazoa parasitemia (Table 1).

Bacteria-killing ability and hemolytic-complement ability are significantly correlated with each other, but nothing else (Table 1). We found no difference between age groups for either measure of immune defence. In contrast, H/L ratio was negatively correlated with scaled-mass index (Table 1) and was lower in juveniles (0.80 ± 0.03) than adults (1.75 ± 0.26).

The most parsimonious Burnham models included constant reporting and encounter probability across both months and years. Monthly encounter probability was 0.024 ± 0.005. Similarly, models indicated no differences in apparent survival probabilities between months, years or time of year an individual was captured. There were differences in survival rates between juveniles and adults. Monthly apparent survival for juveniles was 0.79 ± 0.03, and monthly survival for adults was 0.93 ± 0.01.

**Figure 1:**
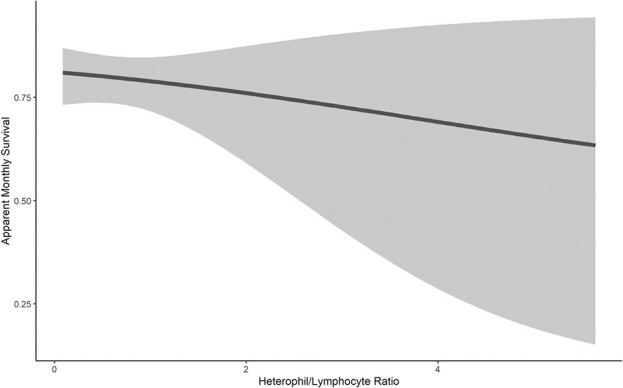
Relationship between model-averaged monthly apparent survival and the ratio of heterophils to lymphocytes at the time of capture in juvenile red-tailed hawks migrating through the Marin Headlands, California (n = 331).

**Figure 2:**
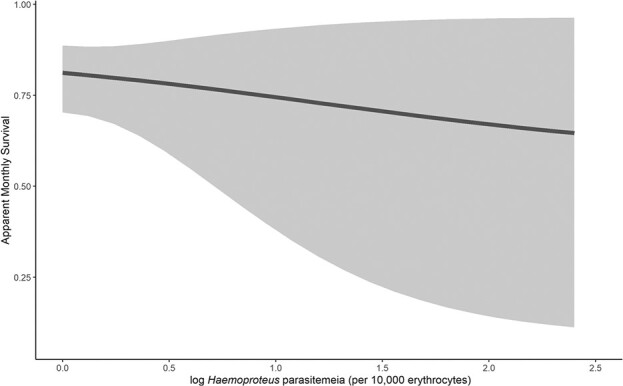
Relationship between model-averaged monthly apparent survival and haemoproteus parasitemia in juvenile red-tailed hawks (n = 331) migrating through the Marin Headlands, California.

We considered all models with a ΔAIC*_c_* < 2 as informative (Table 2). This top set of models included two models. H/L ratio was the most parsimonious predictor of apparent monthly survival across an individual's lifetime, and model-averaged beta estimate did not overlap zero (beta estimate = −0.42 ± 0.22; Σ*ω_i_* = 0.40; Table 2; Fig. 1). *Haemoproteus* parasitemia was also negatively associated with apparent monthly survival across an individual's lifetime (Σ*ω_i_* = 0.32, model-averaged beta was −1.14 ± 0.72; Table 2; Fig. 2). All covariates that were associated with just the month post-capture performed more poorly than models that used covariates to predict survival for the entire lifetime of the individual.

**Table 2:** Survival parameter model results from Program MARK examining the effects of condition on monthly survival in migrating red-tailed hawks (n = 353)

Apparent survival correlates	ΔAIC*_c_*	AIC*_c_* weights	Model likelihood	Parameters	Deviance
Age + H/L	0	0.39	1	7	907.96
Age + Haemo	0.45	0.31	0.80	7	908.40
Age + SMI	2.96	0.09	0.23	7	908.84
Age + BKA	3.00	0.08	0.22	7	910.96
Age + SRBC	3.03	0.07	0.22	7	910.99
Age + H/L (month)	8.08	0.01	0.02	7	916.04
Age	9.47	0.00	0.01	6	919.50
Constant	20.14	0.00	0.00	5	936.32

Age was divided into two groups, juvenile and adult. For all models, *r* and *P* were constant. All models above had constant reporting and encounter probability across both months and years. (month) indicates the covariate only applied to the month following capture. Abbreviations: heterophil/leucocyte ratio (H/L), bacteria-killing ability (BKA), scaled-mass index (SMI), *Haemoproteus* parasitemia (Haemo), and sheep red blood cell lysing ability (SRBC)

## Discussion

Migration may limit population growth for many species because associated energetic costs can force individuals to invest suboptimally in other activities leading, ultimately, to reduced survival. This interaction might, in part, be facultative and dependent on parasite infection (Møller and Szép, 2011). We found that parasitemia of *Haemoproteus* and H/L ratio were both negatively associated with survival over the lifetime of the bird. Higher energetic costs of heavy *Haemoproteus* parasitemia may be difficult for individuals to overcome (Puente *et al.*, 2010; Schoenle *et al.*, 2017), particularly during energetically demanding migration. That the effects of *Haemoproteus* infection lasted for longer than the month post-capture suggests that individuals did not develop a tolerance strategy to high parasitemia by reducing the fitness consequences of the infections (i.e. the infection continued to cause lower survival).

Despite the negative relationship between *Haemoproteus* and survival, we did not find a trade-off between survival and our measures of innate immune function. That is, individuals with higher abilities to fight infection did not survive at higher rates compared to individuals who invested less in constitutive immune defences as predicted, even during just the migratory period (i.e. the month post-capture). Relatedly, we did not find correlations between our measures of immune defences and parasite infections. Together, these results suggest that the immune defences we measured were not regulating the relationship between parasites and survival, and the costs of these immune defences did not scale to individual-level differences in survival as predicted by trade-off theory (Lochmiller and Deerenberg, 2000; Downs *et al.*, 2014).

Although immune defences are costly (Lochmiller and Deerenberg, 2000; Bonneaud *et al.*, 2003), the relative costs depend on the type of immune defence measured (Armitage *et al.*, 2003; Derting and Compton, 2003; Lee, 2006). We measured responses that are part of the constitutive, humoral immune system, a system that primarily uses globular proteins to recognize, bind and remove pathogens (Murphy *et al.*, 2007). Maintenance of constitutive humoral responses is generally less costly than induced cellular immune responses or humoral responses activated during an immune challenge (Demas *et al.*, 1997; Armitage *et al.*, 2003; Derting and Compton, 2003). Furthermore, the facultative response hypothesis predicts, and empirical data support, that allocation trade-offs involving immune defences are often only observed when food is limited (French *et al.*, 2007a, 2007b), a condition that may not be true in our system. Therefore, the environmental conditions experienced by the hawks at the time of measurement and the type of immune defences we measured might have contributed to the lack of an observed trade-off.

We also found no relationship between either measure of immunity and parasitemia. We measured constitutive immune defences in our study. Still, we do not know if our measures reflected baseline or a response to active parasite infections because we do not know the history of the birds. Furthermore, we could not quantify parasitemia or presence/absence for every parasite (Downs and Stewart, 2014). Our measure of immune defences mainly measured aspects of the complement cascade (French *et al.*, 2010; Demas *et al.*, 2011). Typically, both of these measures increase during an active immune infection because detection of an immune challenge causes an upregulation of the complement response (Millet *et al.*, 2007; Wilcoxen *et al.*, 2015; MacColl *et al.*, 2017).

An allocation framework provides two potential, nonmutually exclusive reasons why we did not find a relationship between parasitemia and immunity. First, birds in our study were actively undergoing an energetically expensive activity, migration, and birds may have forgone investing in high complement responses to invest resources into migration despite having active infections. This may be mediated by immunosuppression caused by the energetic demands of migration (Weber and Stilianakis, 2007); migration parallels extreme exercise, which causes immunosuppression (Gleeson, 2004). Alternatively, birds might have invested in a tolerance rather than resistance strategy because of the cost constraint imposed by migration (Sears *et al.*, 2006). In this case, birds would minimize the fitness costs of the parasite infection rather than mounting a large immune response to clear the infection (Råberg *et al.*, 2009). This latter interpretation is supported by the survival modelling, which found that only very high levels of parasitemia led to a decline in survival probability.

H/L ratio was similarly negatively correlated with apparent survival over the course of the entire study. H/L ratio has also been negatively correlated with survival in other avian species, such as great tits (*Parus major*) and pied flycatchers (Lobato *et al.*, 2005; Kilgas *et al.*, 2006) and serves as a proxy for physiological condition (Gross and Siegel, 1983). Our results also support the idea that birds in poor physical condition, as indicated by the scaled-mass index, are under more physiological stress resulting in higher H/L ratios (Hanauska-Bro and Dufty, 1996; Vleck *et al.*, 2000; Suorsa *et al.*, 2004). Together these results suggest that H/L ratio is an informative measure of individual quality in migrating red-tailed hawks.

We found that adults have significantly higher H/L ratios than juveniles, which is common in avian species (Minias, 2019). Although we know little about the ontogeny of the immune system in wild birds, we know that the acquired immune system continues to develop throughout a hawk's lifespan (Dehnhard *et al.*, 2011; Jakubas *et al.*, 2015). Therefore, we would expect H/L ratios to differ between juveniles and adults. However, we do not know if the difference in H/L ratio is strictly due to continuous development of the immune system or differences in physiological condition arising from previous migrations, possibly reproduction or disease exposure (Davis *et al.*, 2008).

Our other measures of individual condition (scaled-mass index) and infection (*Leucocytozoon* parasitemia) were unrelated to monthly survival. Previous work suggests that *Leucocytozoon* often affects reproduction (Merino *et al.*, 2000) and survival (Møller and Nielsen, 2007; Niedringhaus *et al.*, 2018). A possible explanation for our null results is that we may be biased against trapping very sick individuals because they may have been unavailable for capture (Frank, 1996). Given our small sample size and potential for biasing against birds with high loads or highly virulent strains, we might not have been able to pick up small effects on survival caused by *Leucocytozoon*.

We expected to, but did not find, that parasitemia and H/L ratio differ across the migration season because birds migrating later in the season are more likely from the intermountain west than central California (Hull *et al.*, 2009; Briggs *et al.*, 2020). Higher parasitemia may be the result of the costs of longer migration distances, which can result in lower immune activity (Eikenaar and Hegemann, 2016). Consistent with this prediction, red-tailed hawks in this migration pathway and trapped later did have higher parasite prevalence (Ishak *et al.*, 2010). These longer-distance migrants may have higher chances of being exposed to parasites because they encounter both more conspecifics and heterospecifics (Krauss *et al.*, 2010). However, we need more information on parasite vector populations and seasonal distribution to understand the risk of exposure to hematozoa across the annual cycle.

This study informs our understanding of how parasite prevalence, H/L ratio and immune functions relate to each other and survival for migrating western red-tailed hawks, which has implications for understanding the health of this species at an individual and population level. By integrating data about parasites and physiology into survival models, we were able to illuminate both short-term and long-term effects of individual-level traits on apparent survival in this population. Linking individual-level physiology with population vital rates, such as survival, is critical for understanding how interindividual variation could mediate population-level processes, which can have consequences for management decisions (Downs and Stewart, 2014).

## Funding

This work was supported by the GGRO Data Analysis and Publishing Fund and the Gregory Hind Endowment. GGRO is a program of the Golden Gate National Parks Conservancy in coordination with the National Park Service. This is GGRO contribution No. 207.

## Data Availability

The data underlying this article cannot be shared publicly because it is part of a long-term monitoring project. The data will be shared on reasonable request to the corresponding author.
